# Eye Strain a Cause of Nocturnal Enuresis

**Published:** 1895-04

**Authors:** 


### Eye-Strain a Cause of Nocturnal Enuresis.

Dr. Geo. M. Gould reports a number of cases of children who were afflicted with nocturnal enuresis, that were cured by correction of the ocular defect by glasses. In most of the children the involuntary urination was accompanied by many other nervous symptoms, such as night terrors, headaches, chorea, etc., nearly all of which were relieved or cured by glasses that corrected the visual anomaly. Some of the patients had undergone operations and treatment that had extended over years without relief of the trouble.—*Med. News.*

